# Animal Heat

**Published:** 1869-04-01

**Authors:** 


### *Animal Heat.*

BY TSCHESCHICHIN.

The investigations of the author have been made upon rabbits in the laboratory of Dubois-Reymond. He arrives at the following conclusions:

1st.—The spinal cord, containing the circulatory and respiratory centers, acts mediately upon the organic chemical processes, and consequently upon animal heat.

2d.—Section of the spinal-cord results in retardation of the circulation, which induces repletion of the veins by the blood, and in consequence of this, radiation increases, and the general temperature diminishes.

3d.—If the body of the animal be enveloped in bad conductors of heat, the loss of caloric from the surface of the body is diminished; the diminution of temperature may then be diminished or prevented at will; and inversely, the colder the medium in which the animal is placed after the section of the cord, the more rapidly it becomes cold.

4th.—Since the augmentation of the radiation of superficial caloric is due to paralysis of the vessels, and to their repletion with blood, all measures which remove this paralysis retard the radiation and the loss of heat.

5th.—The causes which induce paralysis of the vessels act upon the radiation of caloric, as does section of the spinal cord.

6th.—The cramps, which are produced after the ingestion of certain poisons increase the internal temperature of the bodies of animsls.

7th.—In poisoning by alcohol the general temperature commences to sink immediately after the poisoning.

8th.—Section of the great sympathetic has, upon the general temperature, the same effect as section of the spinal cord.

9th.—Section of the pneumogastric has no appreciable direct influence upon the changes of animal temperature.

10th.—Section of the medulla oblongata at its point of union with the pons-varolü, results in painful febrile phenomena.

11th.—The same febrile phenomena are manifested after the injection of animal fluids in process of putrefaction.

12th.—Physiological experiments and clinical facts confirm the presence of *moderator centers* in the brain.

